# Homology-Free Detection of Transposable Elements Unveils Their Dynamics in Three Ecologically Distinct *Rhodnius* Species

**DOI:** 10.3390/genes11020170

**Published:** 2020-02-06

**Authors:** Marcelo R. J. Castro, Clément Goubert, Fernando A. Monteiro, Cristina Vieira, Claudia M. A. Carareto

**Affiliations:** 1 UNESP—Univ. Estadual Paulista, Departamento de Biologia, São José do Rio Preto, SP 15054-000, Brazil; mrjuradoc@gmail.com; 2 Department of Molecular Biology and Genetics, 107 Biotechnology Building, Cornell University, Ithaca, New York, NY 14853, USA; goubert.clement@gmail.com; 3 Laboratório de Epidemiologia e Sistemática Molecular, Instituto Oswaldo Cruz, FIOCRUZ, Rio de Janeiro 21040-360, Brazil; fam@ioc.fiocruz.br; 4 Laboratoire de Biométrie et Biologie Evolutive, Université de Lyon, Université Lyon 1, CNRS, UMR5558, F-69622 Villeurbanne, France; cristina.heddi@univ-lyon1.fr

**Keywords:** repeatome, *Mariner* family, burst of transposition, dnaPipeTE, *Rhodnius prolixus*, *Rhodnius montenegrensis*, *Rhodnius marabaensis*

## Abstract

Transposable elements (TEs) are widely distributed repetitive sequences in the genomes across the tree of life, and represent an important source of genetic variability. Their distribution among genomes is specific to each lineage. A phenomenon associated with this feature is the sudden expansion of one or several TE families, called bursts of transposition. We previously proposed that bursts of the *Mariner* family (DNA transposons) contributed to the speciation of *Rhodnius prolixus* Stål, 1859. This hypothesis motivated us to study two additional species of the *R. prolixus* complex: *Rhodnius montenegrensis* da Rosa et al., 2012 and *Rhodnius marabaensis* Souza et al., 2016, together with a new, de novo annotation of the *R. prolixus* repeatome using unassembled short reads. Our analysis reveals that the total amount of TEs present in *Rhodnius* genomes (19% to 23.5%) is three to four times higher than that expected based on the original quantifications performed for the original genome description of *R. prolixus*. We confirm here that the repeatome of the three species is dominated by Class II elements of the superfamily *Tc1-Mariner,* as well as members of the LINE order (Class I). In addition to *R. prolixus*, we also identified a recent burst of transposition of the Mariner family in *R. montenegrensis* and *R. marabaensis*, suggesting that this phenomenon may not be exclusive to *R. prolixus*. Rather, we hypothesize that whilst the expansion of *Mariner* elements may have contributed to the diversification of the *R. prolixus*-*R. robustus* species complex, the distinct ecological characteristics of these new species did not drive the general evolutionary trajectories of these TEs.

## 1. Introduction

Transposable elements (TEs) are repetitive DNA sequences capable of replicating and moving among genomes. This fraction of the repeatome [1] is quite variable in eukaryotes, ranging from 4 to 74% of fungus genomes [2] and 3 to 45% of metazoan genomes [3]. This proportion can be even higher in plants, representing up to 85% of the genome in grasses [4]. Despite the documented deleterious effects of TEs at the genomic and phenotypic level [5,6], their prevalence and mobility may lead to structural and functional genomic changes [7,8,9], promoting TEs as powerful evolutionary agents [10].

Based on the hierarchical system proposed by Wicker et al. [11], TEs are classified into two classes depending on their mode of transposition. Class I elements (retrotransposons) such as Long Terminal Repeat retrotransposons (LTRs), LINEs (Long INterspersed Nuclear Elements), and SINEs (Short INterspersed Nuclear Elements) mobilize via RNA intermediates. Class II elements, or DNA transposons, achieve transposition via a DNA intermediate. This class can be further subdivided into subclass 1, which includes the orders TIR (Terminal Inverted Repeat) and Crypton, and subclass 2, which includes the orders Helitron and Maverick.

The abundance of TEs, their genomic distribution, and the chromosomal rearrangements that transposition triggers are often lineage-specific, and this specificity suggests that their activity can be associated with the speciation process, either as a cause or a consequence [12,13]. The multiplication or sudden increase in the copy number of one or several TE families, also called a “burst”, may cause such genomic reconstruction. This phenomenon, which occurs in short periods during the lifecycle of a TE family, has been associated with large evolutionary events and the genesis of new phylogenetic groups [14,15,16,17,18,19,20]. Bursts of transposition maybe also be a common reaction of the genome to rapid changes in external environments. However, other genomic stresses, such as domestication, polyploidy, interspecific and intergeneric hybridization, or even changes in mating systems, are also associated with transposition bursts (reviewed by Belyayev [21]). While TE bursts can contribute to the generation of new biological diversity and eventually lead to speciation, the causal relationship of this association has yet to be investigated. 

To test whether TE bursts contributed to speciation in the *Rhodnius* complex, we compared the repeatomes of three closely related species vectors of the Chagas disease—*Rhodnius prolixus*, *Rhodnius montenegrensis* [22], and *Rhodnius marabaensis* [23]—that recently diverged from their common ancestor and currently exhibit quite different ecologies. 

*Rhodnius prolixus*, which belongs to the complex of cryptic species *R. prolixus*-*R. robustus* [24,25,26,27], is a primary vector of the Chagas disease across northern South America. This zoonotic disease, the fourth most infectious in the Americas, is caused by the protozoan *Trypanosoma cruzi* (Chagas 1909) and is transmitted to humans by haematophagous insects of the Triatominae subfamily [28]. Within this complex, the *Rhodnius* subclade, which includes the three species investigated here, likely represents a late Pliocene–Pleistocene radiation that generated *R. prolixus* in the Orinoco basin and a core Amazon cluster, including *R. montenegrensis* to the southwest and *R. marabaensis* to the southeast [27]. While *R. prolixus* is the main domestic Chagas disease vector in Colombia, Venezuela, and certain areas of Central America, *R. montenegrensis* and *R. marabaensis* are exclusively sylvatic, having relatively little medical relevance, and are distributed throughout the Amazon region [25,29,30].

As currently described in the literature, the repeatome of *R. prolixus* is unusually enriched with Class II elements, which represent 75% of all TEs identified in the assembly. These TEs are mainly represented by the *Tc1-Mariner* superfamily and MITEs (Miniature Inverted-repeat Transposable Elements, derived from Class II—DNA/TIR elements such as *Mariner*) [31]. Most of the *Mariner* sequences are full-length, suggesting that they are or have been active until recently. Moreover, most of the *Mariner* sequences are related to TEs found in seven well-established insect TE subfamilies (*irritans*/*himar*, *mellifera*, *cecropia*, *mauritiana*, *vertumnana,* and *rosa*) in addition to two novel *Mariner* subfamilies, *prolixus1* and *prolixus2*, which exhibit a high number of copies [32]. In the *prolixus1* subfamily, three clades of sequences were identified, one with more than 8,000 copies (clade I) and the two others with 846 (clade II) and 550 (clade III) genomic copies. These three TE clades underwent intense genomic expansions, which is characteristic of a burst of transposition. The most recent common ancestral sequence of *prolixus1* clade I was dated at approximately 1.32 million years ago (mya) and that for clades II and III at approximately 0.67 mya. The oldest period coincides with the proposed time for divergence of *R. prolixus* (~ 1.4 mya) [25].

The low genetic diversity among *R. prolixus* populations [24,25,33,34] led Fernandez-Medina et al. to propose that the burst of transposition of the *pro*lixus1 and *prolixus2 Mariner* subfamilies could have started as a result of the ecological and populational constraints of the species [32]. The authors also suggested that the continuous expansion of these TEs could be associated with the increased adoption of domestic habitats by man, which occurred with the colonization of the Americas [25]. However, the alternative hypothesis, postulating that the bursts of transposition occurred prior to the *R. prolixus*/*R. montenegrensis*-*R. marabaensis* split has not been formally tested and rejected. To test this hypothesis, we annotated the repeatomes of two of the four members of the *R. robustus* cryptic species complex (*R. montenegrensis* and *R. marabaensis*) from the Amazon region and compared them to that of the *R. prolixus*. Our results reveal that the overall TE content of the three *Rhodnius* species is actually three to four times higher than that previously estimated using the assembled *R. prolixus* genome [31] and strongly suggest that the TE *Mariner* transposition burst occurred before the lineage diversification.

## 2. Materials and Methods 

### 2.1. Sequencing Data

The genomes of *R. montenegrensis* and *R. marabaensis* were sequenced using Illumina (150 bp–~30×) *TruSeq Nano paired end* by the GeT-PlaGe, Génopole Toulouse/Midi-pyrénées platform (France). DNA was extracted from three individuals of *R. montenegrensis* (collected in Ouro Preto do Oeste, Rondônia state, Brazil, 10°43′ S 62°15′ W) and two individuals of *R. marabaensis* (collected in Rondon do Pará, Pará, Brazil, 04°54′ S 48°20′ W). To re-analyse the repeatome of *R. prolixus* with the same protocol, we used the raw reads generated during the genome assembly [31], hereafter referred to as *R. prolixus*^1^.

### 2.2. Annotation and Quantification of the Repeat Content

The repeat content of the *R. montenegrensis, R. marabaensis,* and *R. prolixus* genomes was assembled, annotated, and quantified from raw reads using the pipeline dnaPipeTE v1.3 [35]. dnaPipeTE assembles genome repeats from low coverage samplings of raw reads (typically < 0.5X) and annotates TEs by homology using RepeatMasker (www.repeatmasker.org). Eventually, the relative abundance of the discovered repeats is quantified by mapping a random sample of the reads onto the assembled repeats. Accordingly, dnaPipeTE was run with the three species on the low coverage data using the following parameters: -genome_size 702000000 -genome_coverage 0.25 -sample_size 2 -RM_t 0.2 -Trin_glue 2. These parameters ensure that two iterations of the Trinity assembler are performed using independent read sets, sampled at 0.25X each time. Two overlapping reads are needed to bind together the k-mer contigs build during the TE assembly (-Trin_glue 2), and the annotation threshold was set such that 20% of a dnaPipeTE contig (de novo assembled repeat) must be covered by a RepeatMasker hit to keep the annotation (Supplementary Figure S1A–D). 

In the absence of direct measurement of *R. montenegrensis* and *R. marabaensis* genome size, we used the estimated genome size of *R. prolixus* (733 Mb, same strain as in this study) [31]. Preliminary dnaPipeTE analyses were performed using an increasing number of reads per run (up to 1X) before settling on a coverage of 0.25×, as this value maximized the N50 of the assembled genomes. Accordingly, and because of their phylogenetic proximity, a 0.25× coverage was also chosen to analyse the raw reads available for *R. prolixus* (*R. prolixus*^1^).

In addition to these assembly-free estimates, and to provide a point of comparison with Mesquita et al. [31], we also re-analysed the genome of *R. prolixus* based on its assembled sequence. While the initial TE description in *R. prolixus* was solely based on homology methods [31], we assembled here the repeats without prior knowledge, proposing a new usage of dnaPipeTE. Indeed, dnaPipeTE initially requires single-end short reads (typically unassembled Illumina reads); thus, the genome assembly of *R. prolixus* (702 Mb)—hereafter named *R. prolixus*^2^—was first fragmented into short reads using ART [36]. To simulate single-end HiSeq 2500 150 bp reads with no sequencing errors, the parameters were “-ss HS25 -l 150 -f 10 -ef”; dnaPipeTE was then run on the simulated reads using the same parameters as chosen for the newly sequenced specimens.

### 2.3. Comparisons of Shared TE Families

Because TE copies of a given family vary in sequence, structure, and abundance, dnaPipeTE often produces multiple contig per family in order to reflect this diversity (Supplementary Figure S1A,B). Thus, for each possible pair of species (three comparisons), the contigs produced by dnaPipeTE in each species were concatenated and clustered (Figure S1C) to (i) reconstruct TE families and (ii) identify the one shared by the pair (clusters with contigs from both species). We used CD-HIT-EST (version 4.6.1) [37] with the parameters -aS 0.8 -c 0.8 -G 0 -g1, ensuring to group sequences with at least 80% identity in an alignment representing at least 80% of the shortest sequence (Supplementary Figure S1C, criteria delineating a TE family according to Wicker et al.) [11]. Then, repeat clusters (putative TE families) were annotated and quantified using the file “read_per_component_and_annotation” generated by dnaPipeTE in each species. This file contains for each dnaPipeTE contig its quantification in a random sample of reads as well as the TE annotation given by RepeatMasker, if available. Finally, the proportions of TE families in each genome were inferred based on the base pair count of each species in a cluster (Supplementary Figure S1D). The size (as genome percentage), species distribution, and sharing of TE families was then compared between species. For each identified shared TE family, we calculated the linear regression (Minitab 18) to model the relationship between the proportions of shared TEs between the two genomes (Figure S1E). In addition, the 95% two-sided confidence intervals where calculated to evaluate the robustness of the correlations. 

## 3. Results

**Figure 1:**
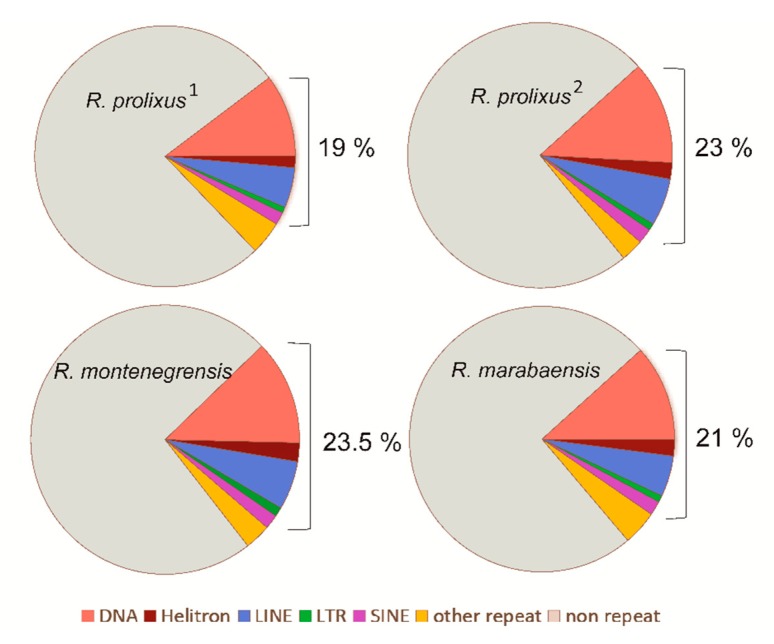
Relative proportions of the main repeat classes in genomes of *R. prolixus*^1^, *R. montenegrensis*, *R. marabaensis* (annotation from raw genomic reads), and *R. prolixus*^2^ (annotation from fragmented reads from the assembled genome).

We first analysed the variation in the TE content of three closely related *Rhodnius* species by processing unassembled low-coverage samples of short sequencing reads. The three species have similar proportions of TE-derived reads in their genomes: with 19% (*R. prolixus*^1^), 23.5% (*R. montenegrensis*), and 21.2% (*R. marabaensis*) of the sampled reads mapping to the assembled TE contigs Figure 1). In the three genomes, the most abundant TEs were annotated as DNA transposons (Class II), corresponding to 10.2% (*R. prolixus*^1^), 12.6% (*R. montenegrensis*), and 11.7% (*R. marabaensis*) of the reads or 61.4% (*R. prolixus*^1^), 63% (*R. montenegrensis*), and 66.3% (*R. marabaensis*) of the total TEs annotated (Supplementary Table S1). LTR retrotransposons were the least represented class of TEs (min: 0.8%, *R. prolixus*^1^ and *R. marabaensis*; max: 1.1%, *R. montenegrensis*). All TE classes considered, these inferred TE proportions (19–23.5%) are much higher than previously reported in *R. prolixus* (5.6%) [29]. Consistent with the results obtained from raw reads, the reanalysis of the *R. prolixus* assembly (*R. prolixus^2^* dataset) obtained from the fragmented genome assembly, corresponded to 23% (Figure 1, Supplementary Table S1). Of these TEs, the major portion also corresponds to DNA transposons (12.55%), and the smallest portion to LTR retrotransposons (0.8%). The other fractions of the genome are given in Figure 1 and Table S2.

### 3.1. Proportions and Divergence of TE Superfamilies in Rhodnius Species

**Figure 2:**
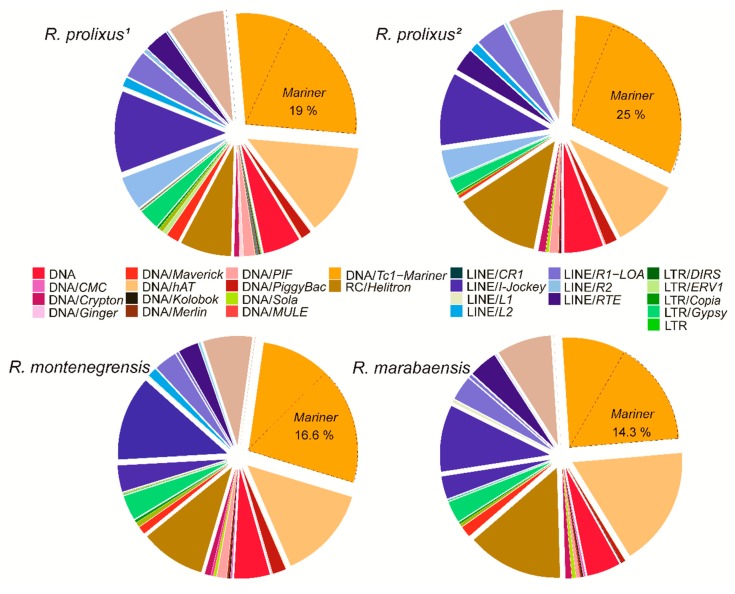
Relative repeatome proportions of main superfamilies from dnaPipeTE annotation (BLASTN with 0.25_ genome coverage) for *R. prolixus* (^1^ and ^2^) and *R. montenegrensis* and *R. marabaensis*).

Figure 2 shows the proportions of superfamilies in relation to the total repeatome and the total TE content in the four genomes. The most abundant superfamily is *Tc1-Mariner*, varying from 5.27% in *R. prolixus*^1^ to 7.0% in *R. prolixus*^2^, corresponding to 27.9% to 30.4% of the total TEs, respectively. Among the *Tc1-Mariner* elements, *Mariner* is the most abundant family, corresponding to 14.3% (*R. marabaensis*) to 25% (*R. prolixus*^2^) of the total TEs in the genome. In Class I, *Jockey* (non-LTR retrotransposon) is the most frequent superfamily, representing only 2.1% (*R. montenegrensis*) to 2.94% (*R. marabaensis*) of the genome and 9.77% to 12.48% of the total TEs, respectively (Table S1). The most abundant TE families identified (> 0.10%) in the three genomes are presented in Table S3.

**Figure 3:**
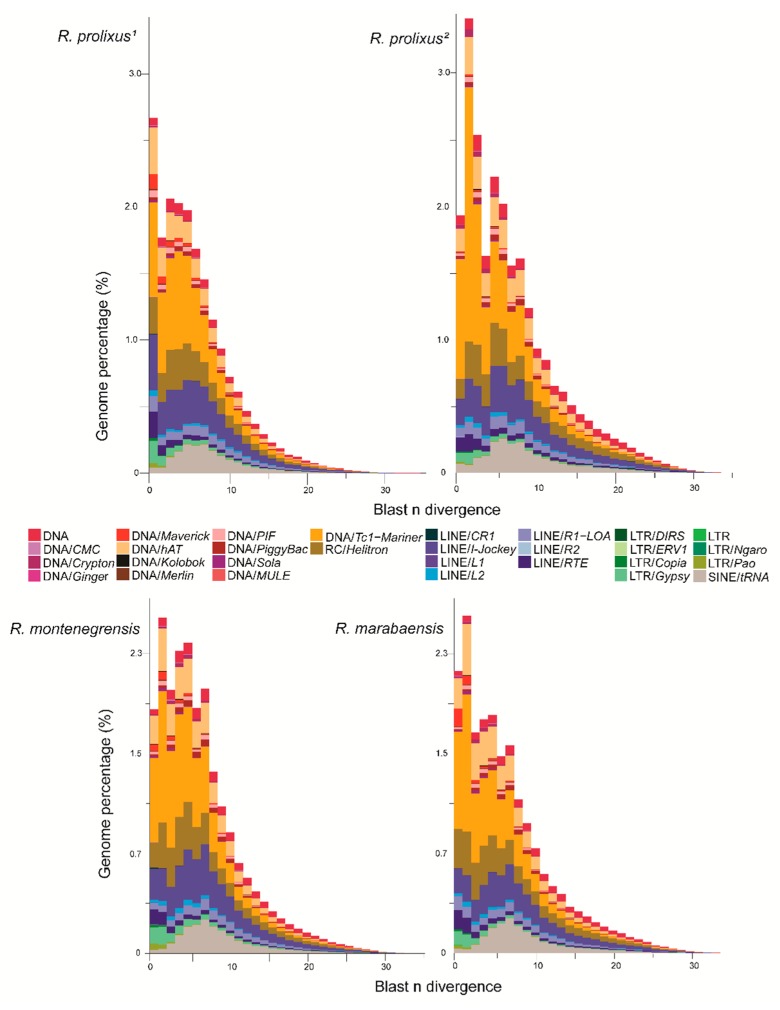
Distribution of TE ages between the *Rhodnius* genomes. For each species, the nucleotide divergence from BLASTN is calculated between a repeat read and the contig, where it matches the dnaPipeTE assembly. *R. prolixus^1^*: annotation from raw genomic reads; *R. prolixus^2^* (annotation from fragmented reads from the assembled genome).

The relative distribution of superfamily ages, based on the divergence between sampled reads and the annotated dnaPipeTE contigs are presented in Figure 3. These landscapes suggest that the TEs evolved similarly in the three species. The results suggest that DNA transposons underwent a recent burst (< 1–2% divergence) following the expansion of SINEs, which show an older hallmark of activity (< 5% divergence). Among the DNA transposons, the *Mariner* family, which is the most abundant, presents reads from TE copies that are highly similar to the annotated contigs, suggesting a recent burst of transposition. While less abundant, LTR retrotransposons seem to be very recent (0–2% divergence). Finally, the landscape of LINEs is consistent with the maintenance of a relatively constant rate of transposition throughout recent evolution, with the noticeable exception of a member of the LINE/RTE clade, which appears to have recently increased in copy number.

### 3.2. Relative Proportions of TE Families Shared between the Rhodnius Species

**Figure 4:**
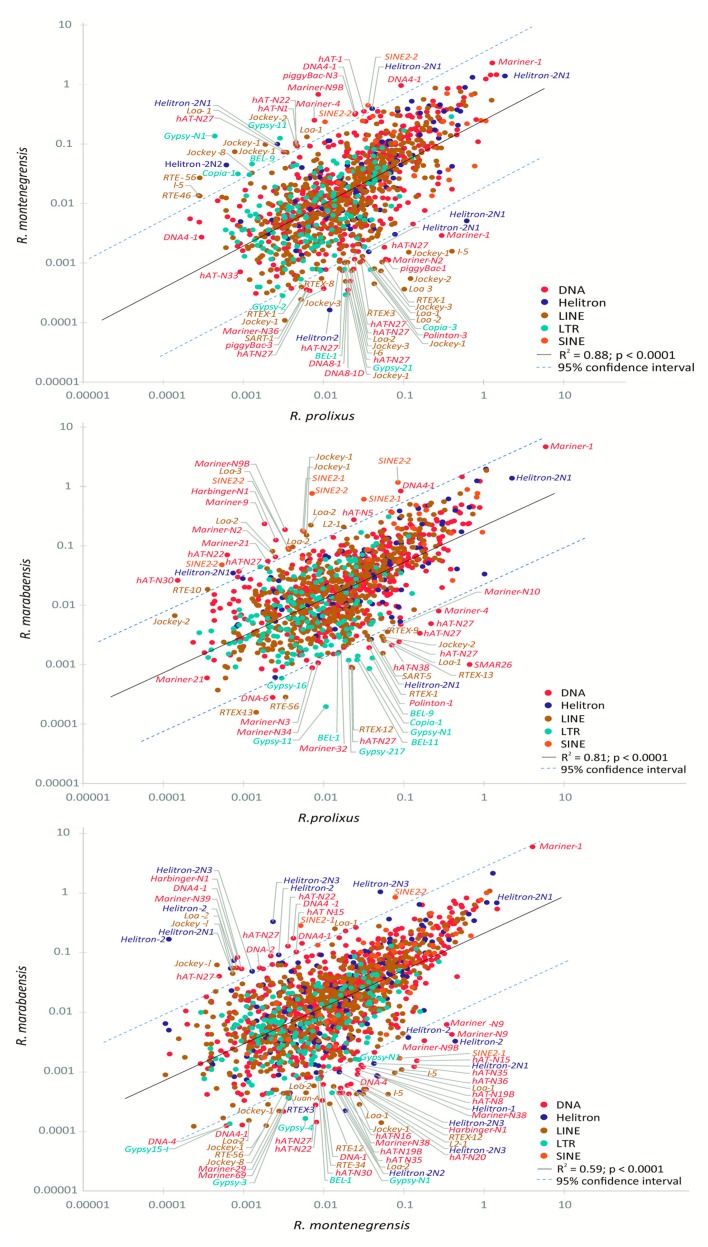
Scatter plot and analysis of linear regression of the relative genome proportions of shared TE families between the *Rhodnius* genomes in terms of genome percentage (log10 scale). Each dot represents a shared TE family, defined by the highest BLAST hit between the TE family reference contig of each species.

In agreement with the above-cited results, the comparative analyses carried out among the three *Rhodnius* genomes revealed that, based on the pattern of presence/absence, a high percentage of TE families are shared between pairs of genomes: ~ 74% for *R. prolixus*^1^—*R. montenegrensis*, ~ 76% for *R. prolixus*^1^—*R. marabaensis* and ~ 80% for *R. montenegrensis*—*R. marabaensis*. In general, most families have similar genomic proportions between species (mainly DNA transposons and non-LTR retrotransposons), including the DNA transposons *Mariner-1* (~2%) and *Helitron2-N1* (~1.5%) (Figure 4, Figure S2). Correlation analyses showed positive, highly significant relationships between the proportions of the shared TE families. However, we also found several shared families with a high degree of variation in proportions between genome pairs, as depicted in the fitted line plot of the linear regression, above and below the 95% confidence intervals (Table S4). 

**Figure 5:**
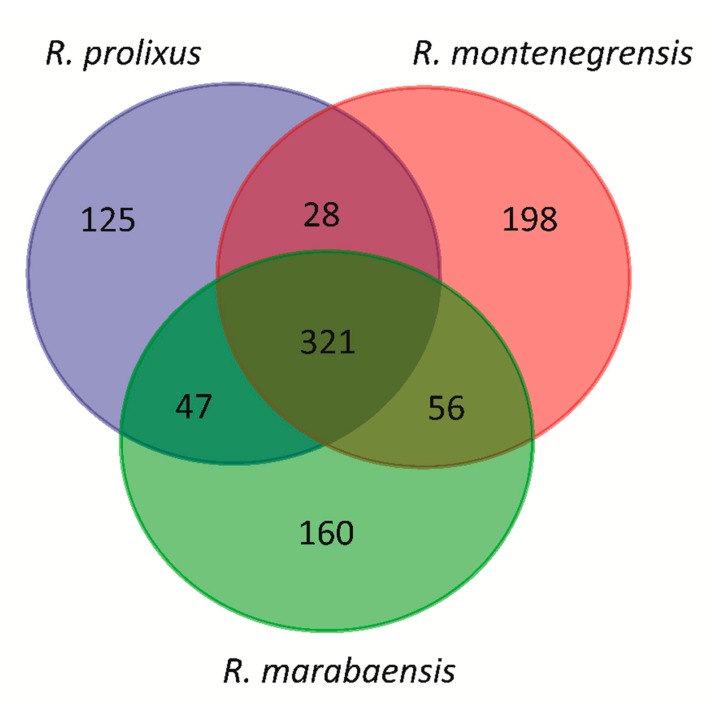
Venn diagram of the distribution of TE families in *R. prolixus*, *R. montenegrensis,* and *R. marabaensis* genomes. Overlapping regions of the Venn diagrams indicate families’ share between the three or two pairs of genomes as well as unique families in each genome.

According to a Venn diagram, three aspects stand out from the repeatome content shared by the three species (Figure 5). First, 321 families are detected in all three species, which make up between 18% (*R. prolixus*) and 22.9% (*R. montenegrensis*) of the genomic content and between 97.6% (*R. marabaensis*) and 98% (*R. marabaensis*) of the detected TEs. Second, there are families only found in two species, as for example 47 families shared between *R. montenegrensis* and *R. marabaensis*, which correspond between 0.06% and 0.08% of the genomic content and 0.22% and 0.40% of the TE content, respectively. Third, there are families unique to each species, which account for only small proportions of the three genomes, such as in *R. marabaensis*, 0.10% of the genomic content and 0.46% of the TE content (Table S5). We highlight the families’ share between two genomes or unique for each one; however, they only represent a very low percentage compared to the families found in the three genomes. In addition, and due to the dnaPipeTE strategy based on read sampling, the absence of a given family in a genome may not mean total absence, but rather a low number of copies that prevented their detection. Similarly, the variations in abundance—even dramatic—between members of low frequency families could be only mirroring the variance due to the sampling method.

## 4. Discussion

Repeatomes of *R. prolixus, R. montenegrensis* and *R. marabaensis* were assembled and annotated de novo from unassembled reads in order to determine whether these closely related lineages underwent shared or independent bursts of *Mariner* elements than those previously reported in *R. prolixus* [31,32]. First, our comparative analysis of the three species’ repeatomes reveals that the TE contents of *Rhodnius* species complexes have been largely underestimated. Indeed, the overall TE content of the three species (~20%) was found to be three to four times higher than the 5.6% previously reported [31]. This previous analysis—performed on an assembled genome—was only based on sequence homology, which is likely to miss unknown and divergent TE families (typically > 30% divergence due to the limitation of the Blastn algorithm) as well as lineage-specific TE subfamilies [38]. In addition, TE-rich genomes are often challenging to assemble, ending up with a high level of fragmentation or collapsing of repetitive regions, and consequently the underestimation of the TE content. This is particularly true when the repeatome is recent and large [39], as observed here.

The three species display very similar TE landscapes, with comparable distributions of families, both quantitatively and qualitatively. In all three species, including our re-annotation of the *R. prolixus* assembly, the repeatome is predominantly composed of Class II elements (~ 65%) including *Tc1/Mariner* (~27%) and *Helitron* (~ 10%). Class I elements represent approximately 35% of each species’ repeatome (with the most abundant being members of LINE superfamilies *Jockey*: ~ 11%, *Loa*: ~ 3.5% and *RTE*: 1% as well as LTR retrotransposons of the *Gypsy* superfamily: ~ 3.5%). These proportions approach those previously reported [31,40], where 75% the repeatome of *R. prolixus* was thought to be made of Class II elements, including 50% made of the *Tc1/Mariner*, *hAT* and *Helitron* superfamilies. The remaining 25% was attributed to Class I, of which 15.5% was represented by members of the superfamilies *Jockey*, *Loa*, *RTE* and *Gypsy* [31,40]. 

The unexpected amount of annotated TEs found in the repeatomes of *Rhodnius ssp.* may be even greater as non-autonomous MITEs (Miniature Inverted-repeat Transposable Elements)—usually challenging to annotate due to their lack of coding sequence—were not specifically searched for. MITEs are small (typically ~100-600 bp) non-autonomous DNA transposons that are highly abundant in several plant and animal genomes, most likely originating from the accumulation of internal deletions of autonomous DNA transposons [41]. One limitation of dnaPipeTE in annotating MITEs is that most are absent from the reference library (Repbase), with the closest homologs being the terminal inverted repeats (TIRs) from their related DNA transposons. According to Mesquita et al., the genome of *R. prolixus* could harbour 0.5% MITEs [31]. Moreover, Zhang et al. reported that *R. prolixus* MITEs are derived from five DNA transposon superfamilies (*Sola*, *hAT*, *Ginger,* and *Tc1/Mariner*) [42], and Filée et al. identified eight clusters of sequences with characteristics of MITEs, four of which were associated with *Mariner* elements present in the genome [40]. As families of *Sola*, *hAT*, *Ginger,* and *Tc1/Mariner* are abundant in the annotation we performed, it is likely that part of the TEs we annotated as Class II with dnaPipeTE (~ 65% of the repeatome) are actually MITEs. To address this issue, homology sequence analyses can be performed on the dnaPipeTE contigs to recover the known MITEs sequence of *R. prolixus*.

The prevalence of Class II elements in *Rhodnius ssp.* may be a feature of this genus, considering that most reported insects’ repeatomes so far have usually a higher abundance of Class I than Class II elements, as seen in *Bombyx mori* Linnaeus, 1758 (Bombycidae, 80%) [43], *Drosophila* Fallén, 1823 (Drosophilidae, 67% to 93%) [44], *Tribolium castaneum* Herbst, 1797 (Tenebrionidae, 63%) [45] or *Anopheles gambiae* Giles, 1902 (Culicidae, 66%) [46]. However, regardless of the relative proportions of these classes in insects, the *Tc1/Mariner*, *hAT* and *Helitron* superfamilies are highly prevalent, as demonstrated by comparative genomic studies of 257 eukaryotic species, including animals, plants, and fungi [47]. A credible explanation for this pattern involves the high rate of horizontal transfer of members of these superfamilies, along with the absence of host genomic defences such as pi-RNAs, which otherwise act in *trans* to silence TE activity [48].

Among the representatives of the *Tc1/Mariner* superfamily in *Rhodnius* complexes, the most abundant family is *Mariner* (~ 17%), consisting mainly of three clades (*Mariner-1_RPr*: ~ 11%, *Mariner-N9_RPr*: ~ 3%, and *Mariner-N9_RPr*: ~ 1%) (Table S3) that collectively account for more than 90% of *Mariner* superfamily copies. In addition, we observed very low, or even no divergence between reads belonging to different genomic copies and their consensus sequence (dnaPipeTE contigs). This observation is typical of bursts of TE families [32,40], in which the number of copies increases faster than their removal or degradation [49,50]. Fernandez-Medina et al. formulated the hypothesis that transposition bursts could be associated with the speciation of the *R. prolixus*, in an attempt to explain the expansion dynamics in these three transposable element clades [32]. Our results clearly indicate that the *Mariner* family bursts seen in *R. prolixus* are not exclusive to this species. Rather, we propose the parsimonious hypothesis that these TE expansions occurred earlier in the ancestor of the *R. prolixus*-*R. robustus* cryptic species complex. Our results also show that despite their distinct ecological characteristics, the new species did not show evidence of different evolutionary TE dynamics. However, the amplification of the *Mariner* elements may have provided some of the molecular variation that led to diversification of the *R. prolixus*-*R. robustus* complex ancestor and adaptation to new environments. Indeed, the multiplication of TE families can mediate genomic rearrangements that can alter gene structure or expression by inserting into or near exons, introns, or regulatory regions [7,8,9,10], thus driving genomic diversity and lineage-specific innovation [51]. Further studies are needed to broaden our understanding of the role of *Mariner* elements and bursts of transposition in the evolution of these species.

In addition, the three genomes compared revealed three important characteristics required to understand the evolutionary dynamics of their repeatomes. First, the size (in genome percent) of the TE families shared between genomes is highly correlated, both at high (e.g., *Helitron-2N1*, *Mariner-1*) and low abundance (e.g., *Nimb*, *RTE*). Second, we observed that several identical families are present in very different proportions between pairs of genomes, i.e., high in one and low in the other genome (e.g., *Helitron-2, Jockey-1*). Third, some families seem unique to a species, but this event is only observed for families with very low proportion estimates (0.45% and 1.86%). Thus, we cannot exclude the possibility that the absence of a particular TE family in a given species is in fact the result of a methodological bias (sampling bias) towards families with high copy numbers. 

The three scenarios above fit the “life-cycle of TEs” [52]. In this hypothesis, a new TE copy is introduced into a naïve genome by horizontal transfer (HT). The rate of occurrence of HT depends on several factors, such as invasiveness, characteristics of the host genome, or environmental factors [53]. An interesting feature of the *Mariner* elements is that they are involved in numerous HT events. Wallau et al. [54] reported HT events for 24 different lineages of *Mariner* in *Drosophila* species. In *R. prolixus*, Filée et al. detected nine HT events of *Mariner* elements, most involving insects such as *Dendroctonus ponderosae* Hopkins, 1902, *Scolia oculata* (Matsumura, 1911), *Bombus terrestris* (Linnaeus, 1758), *Glossina pallidipes* (Austen, 1903)*,* and *Drosophila sp.* [40]. Following TE introduction, the cycle is characterized by two events: (i) reduction of element mobility due to the accumulation of defective copies and (ii) establishment of epigenetic mechanisms of silencing by the host genome. In addition, the final phase, which can last for millions of years, is called senescence, in which the transpositional activity is reduced or suppressed, leading to a decrease in functional copies due to the combined effect of point mutations, excision, and purifying selection. The independent activity of the TEs in each family can explain the occurrence of shared and exclusive families as well as the variation of their relative proportions in the studied genomes.

## 5. Conclusions

In summary, the repeatomes of *Rhodnius* species in general and the *Mariner* family in particular represents an excellent model to study the evolutionary cycle of TEs. The comparable proportions of annotated TEs in the three species suggest that stochastic events and/or different selective pressures acting during speciation did not affect the general evolutionary routes of TEs. Alternatively, they might have provided the molecular basis that led to the radiation of the *R. prolixus*-*R. robustus* species complex. Moreover, the original dynamics of *Mariner* and other DNA transposons may explain their success in *Rhodnius*. We believe that the data and interpretations given here will provide a basis for future studies aimed at understanding the role played by transposable elements during adaptation.

## Supplementary Materials

The following are available online at https://www.mdpi.com/2073-4425/11/2/170/s1, Table S1: Distributions of TE superfamilies in the genomes of *Rhodnius* species, Table S2: Number of base pairs (bp) and proportion (%) of repetitive sequences annotated using dnaPipeTE in the genomes of *Rhodnius* species, Table S3: Most abundant TE families identified (> 0.10%) in the genomes of *Rhodnius* species, Table S4: Pairwise comparisons of specific TE families with the highest variation between genomes of *Rhodnius* species, Table S5. Shared and not shared TE families identified in the genomes of *Rhodnius* species. Figure S1: dnaPipeTE analysis and comparison of shared TE family content, Figure S2: Scatter plot and analysis of linear regression of the relative genome proportions of shared TE families between the *Rhodnius* genomes in terms of genome percentage.
